# CaAl-LDH-Derived High-Temperature CO_2_ Capture Materials with Stable Cyclic Performance

**DOI:** 10.3390/molecules30153290

**Published:** 2025-08-06

**Authors:** Xinghan An, Liang Huang, Li Yang

**Affiliations:** 1 Engineering Research Center for Water Pollution Source Control & Eco-Remediation, College of Environmental Science and Engineering, Beijing Forestry University, Beijing 100083, China; an18502285171@163.com; 2 State Key Laboratory of Efficient Production of Forest Resources, Beijing Forestry University, Beijing 100083, China; 3 State Key Laboratory of Environmental Criteria and Risk Assessment, Chinese Research Academy of Environmental Science, Beijing 100012, China

**Keywords:** CO_2_ capture, calcium looping, CaO-based sorbents, layered double oxides, cyclic stability

## Abstract

The urgent need to mitigate rising global CO_2_ emissions demands the development of efficient carbon capture technologies. This study addresses the persistent challenge of sintering-induced performance degradation in CaO-based sorbents during high-temperature CO_2_ capture. A novel solvent/nonsolvent synthetic strategy to fabricate CaO/CaAl-layered double oxide (LDO) composites was developed, where CaAl-LDO serves as a nanostructural stabilizer. The CaAl-LDO precursor enables atomic-level dispersion of components, which upon calcination forms a Ca_12_Al_14_O_33_ “rigid scaffold” that spatially confines CaO nanoparticles and effectively mitigates sintering. Thermogravimetric analysis results demonstrate exceptional cyclic stability; the composite achieves an initial CO_2_ uptake of 14.5 mmol/g (81.5% of theoretical capacity) and retains 87% of its capacity after 30 cycles. This performance significantly outperforms pure CaO and CaO/MgAl-LDO composites. Physicochemical characterization confirms that structural confinement preserves mesoporous channels, ensuring efficient CO_2_ diffusion. This work establishes a scalable, instrumentally simple route to high-performance sorbents, offering an efficient solution for carbon capture in energy-intensive industries such as power generation and steel manufacturing.

## 1. Introduction

The global challenge of CO_2_ emissions has reached critical severity. According to the Intergovernmental Panel on Climate Change (IPCC), atmospheric CO_2_ concentrations in 2024 surged at an unprecedented rate, nearly doubling the threshold set by international climate agreements. The continuously rising concentration has led to global warming, frequent extreme weather events, and intensified ocean acidification. The resulting El Niño phenomenon has further weakened the carbon sink capacity. Despite progress in energy transition and carbon mitigation efforts, fossil fuels remain the dominant contributors to global emissions, with energy-intensive sectors like steel and cement production being under mounting decarbonization pressure. While renewable energy deployment has expanded, persistent technological limitations in energy storage and carbon cycle efficiency hinder its full potential for emission reduction [1]. In this context, carbon capture, utilization, and storage (CCUS) has emerged as an indispensable cornerstone in climate change mitigation strategies [2]. Among these, CO_2_ capture serves as an essential prerequisite for subsequent utilization and storage [3].

At present, amine scrubbing represents the commercially mature pathway for industrial CO_2_ capture. However, its prohibitive costs, poor operational stability, and limited service life have posed substantial barriers to widespread industrial adoption [4]. In industrial fields such as the cement, steelmaking, and thermal power generation fields, the exhaust gases produced during the production process often contain high-temperature CO_2_. If traditional adsorption methods are used to capture such gases, complex cooling treatment of the high-temperature exhaust gas is required in advance, which inevitably leads to energy loss. Solid adsorption materials capable of directly capturing high-temperature CO_2_ have become a research hotspot due to their ability to break through the energy consumption bottleneck caused by cooling in the adsorption process. Currently, high-temperature adsorbents such as calcium oxide (CaO) [5], alkali metal zirconates [6], and lithium silicate (Li_4_SiO_4_) [7] have attracted significant research interest due to their remarkable high-temperature applicability and CO_2_ capture performance. The CaO-based looping (CaL) process, utilizing CaO as an adsorbent, has garnered extensive attention due to its low cost, rapid adsorption/desorption kinetics [8], and high CO_2_ capture capacity (theoretical value 0.786 g_CO2_/g_CaO_) [9].

The CaL process is established as a viable technology for industrial implementation owing to the CaO precursor’s abundant sources, low cost, non-corrosiveness, and environmental friendliness [10]. However, the CO_2_ adsorption capacity inevitably declines rapidly due to sintering and attrition issues, making the capture capability during cyclic carbonation/calcination reactions particularly critical. Notably, the temperatures in these cyclic reactions typically range from 650 to 900 °C [11], which are significantly higher than the Tammann temperature of CaCO_3_ (approximately 533 °C) [12]. This temperature condition leads to sintering on the CaCO_3_ surface, thereby inhibiting CO_2_ desorption and deteriorating the adsorption performance of CaO [13]. Notably, pore blockage by newly formed CaCO_3_ layers triggers an abrupt transition from rapid to slow carbonation kinetics, owing to the molar volume disparity between CaO (16.79 cm^3^/mol) and CaCO_3_ (36.93 cm^3^/mol) [14]. The molar volume of CaO is less than half that of CaCO_3_, causing immediate structural hindrance as the denser CaCO_3_ phase accumulates to obstruct diffusion pathways during the reaction [4].

To address this challenge, researchers have explored strategies including modifying granulation methods [15], incorporating metal dopants [16], or introducing organic acids to mitigate CaO sintering. Among these, doping CaO with inert materials featuring high Tammann temperatures as support matrices has emerged as a widely validated approach [17]. For instance, materials such as CuO [18], K_2_CO_3_ [19], Al_2_O_3_ [20], and MgO [21] have been shown to effectively retard sintering by virtue of their thermal stability, maintaining the porous architecture of CaO-based sorbents. However, achieving intimate nanoscale mixing between the active CaO phase and the inert support material remains challenging with conventional methods, often limiting the effectiveness of sintering suppression and cyclic stability enhancement. This limitation has prompted the adoption of layered double hydroxide (LDH) precursor strategies, which enable atomic-level mixing for fabricating superior calcium-based composite adsorbents [22]. The general chemical formula of LDH is expressed as [M^2+^_1−x_M^3+^_x_(OH)_2_]^x+^ X^n−1^_x_/_n_·mH_2_O, where M^2+^ and M^3+^ denote layer metal cations (typically Mg^2+^, Zn^2+^, Ni^2+^, or specific Ca^2+^ ratios for M^2+^; Al^3+^, Mn^3+^, etc., for M^3+^), X^n−^ represents interlayer anions (e.g., NO^3−^ and CO_3_^2−^), “x” signifies the molar ratio M^3+^/(M^2+^ + M^3+^), and “y” indicates the quantity of interlayer water molecules, offering a promising solution due to their intrinsic atomic-level ordered arrangement of metal ions [23]. This atomic-level dispersion inherent in LDH precursors enables the formation of highly homogeneous composites upon calcination [24]. Consequently, LDHs have been extensively investigated as precursors for CO_2_ capture materials owing to their ability to yield oxides with a large specific surface area, high porosity, excellent stability, and tunable surface chemistry [25,26,27]. Upon calcination at elevated temperatures, LDHs undergo structural reconstruction, forming layered double oxides (LDOs) with a characteristic “structural memory effect” [28]. Compared to their LDH precursors, LDOs demonstrate significantly enhanced CO_2_ adsorption performance [29]. This improvement is widely attributed to their large specific surface area, high porosity, excellent stability, and low cost. Furthermore, LDOs possess abundant surface hydroxyl groups, which actively participate in reactions with CO_2_ [30,31]. For example, MgAl-LDOs and CoAl-LDOs have demonstrated significant potential in this field, benefiting from the structural memory effect and enhanced CO_2_ chemisorption capacity of their derived LDOs [22,32].

Building upon this foundation, this study focuses on CaAl-LDH for high-temperature CO_2_ capture applications. While CaAl-LDH-derived oxides (CaAl-LDOs) exhibit exceptional cyclic stability [33,34,35], their inherent CO_2_ adsorption capacity remains fundamentally limited compared to pristine CaO. To overcome this limitation and synergistically combine high capacity with superior stability, an innovative synthetic strategy that incorporates an organic calcium source (calcium acetate) within the CaAl-LDH precursor framework was developed. This approach leverages the precise compositional tunability of LDH and the structural advantages imparted by the LDH/LDO matrix [36], while the organic precursor is anticipated to enhance the porosity and reactivity of the resulting CaO phase within the composite. The resulting CaO/CaAl-LDO composites are designed to exhibit both excellent cyclic stability and enhanced CO_2_ adsorption activity. Moreover, a novel solvent-nonsolvent strategy was present to enable homogeneous nanoscale integration of binary or multicomponent mixtures. This technique requires minimal instrumentation and offers operational simplicity, demonstrating significant potential for scalable implementation. A composite material through the integration of CaO with CaAl-LDH was prepared, where the CaAl-LDH component serves as a CO_2_ adsorbent while simultaneously functioning as a structural stabilizer to mitigate CaO sintering via structural confinement effects. The resultant CaO/CaAl-LDH composites synergistically integrate the high CO_2_ uptake capacity of CaO with the exceptional cyclic stability inherent to CaAl-LDO derivatives. TGA was employed to characterize the adsorptive capacity and cyclic stability of the samples. Comprehensive physicochemical characterizations were conducted for all samples to elucidate the underlying mechanisms responsible for the performance discrepancies among the adsorbents.

## 2. Results and Discussion

### 2.1. The CO_2_ Capture Performance of Layered Double Hydroxide

MgAl-LDH and CaAl-LDH typically adopt petal-like morphologies, characterized by high specific surface areas and unique three-dimensional layered structures [37]. These attributes enable their function as effective support materials to isolate CaO particles, thereby suppressing thermal sintering and agglomeration. Furthermore, the Mg and Al constituents in MgAl-LDH and CaAl-LDH correspond to common additive components; their high-temperature calcination derivatives exhibit elevated Tammann temperatures, thereby enhancing cyclic stability of composite materials.

**Figure 1:**
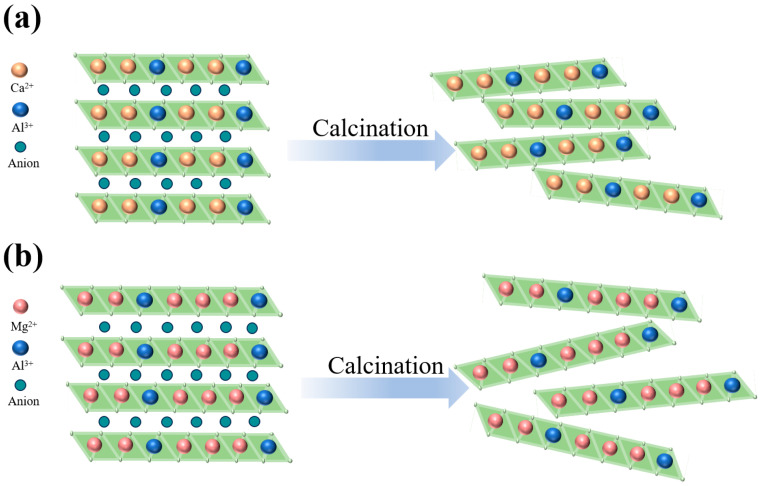
Schematic diagram of (**a**) Ca_2_Al_1_-LDH and (**b**) Mg_3_Al_1_-LDH calcination to produce LDOs.

For inert support materials, thermal stability and anti-sintering ability are the most important features, which ensure that they can effectively prevent the sintering of CaO and remain stable during the cycle. During calcination, hydrotalcite-like compounds undergo stepwise structural evolution. Interlayer anions (e.g., CO_3_^2−^ and NO^3−^) and hydroxyl groups decompose thermally, releasing gaseous products (primarily CO_2_ and H_2_O). This decomposition triggers complete collapse of the layered structure, resulting in stable metal oxide phases, as shown in Figure 1. The resulting layered double oxide (LDO) loses the characteristic layered hydroxide architecture. Instead, the LDO adopts a densely packed crystalline framework comprising metal cations coordinated with oxygen anions. Concurrently, rapid gas evolution during calcination generates extensive microporous and mesoporous networks within the LDO matrix, substantially enhancing specific surface area and developing hierarchical porosity [38,39]. Furthermore, the formation of abundant unsaturated metal coordination sites and oxygen vacancies on LDO surfaces significantly enhances CO_2_ chemisorption capacity compared to the precursor LDH material [28].

**Figure 2:**
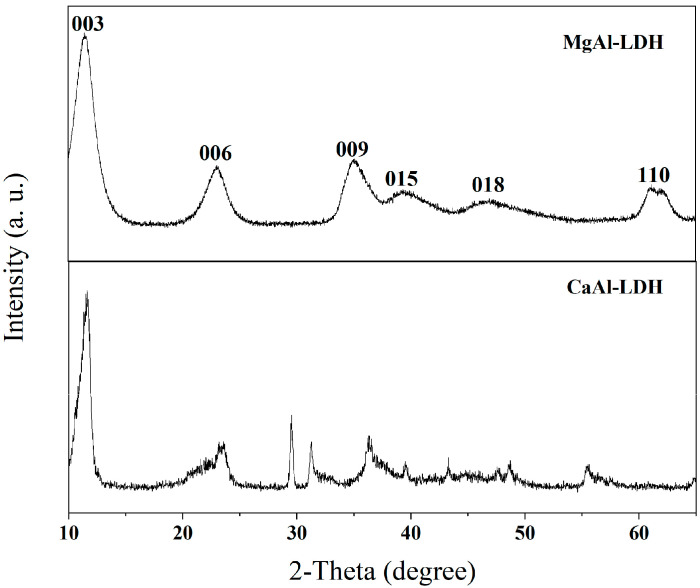
XRD patterns of CaAl-LDH and MgAl-LDH.

XRD characterization was performed to investigate the crystalline structure of as-synthesized MgAl-LDH and CaAl-LDH, with the diffraction patterns presented in Figure 2. The XRD analysis demonstrates that MgAl-LDH displays well-defined basal reflections at 2θ values below 30°, corresponding to the (003), (006), and (009) planes, which are characteristic of the layered double hydroxide structure. The XRD patterns exhibit well-resolved diffraction features with no observable impurity phases, confirming high phase purity. While CaAl-LDH displays marginally broader peaks due to lattice strain induced by the larger ionic radius of Ca^2+^ compared to Mg^2+^, both materials demonstrate stable baseline profiles with no evidence of CaCO_3_, Al_2_O_3_, or other crystalline impurities. These results indicate that synthesized MgAl-LDH and CaAl-LDH possess phase-pure composition, well-ordered layered structures, and XRD-detectable purity consistent with the structural model of LDH materials.

**Figure 3:**
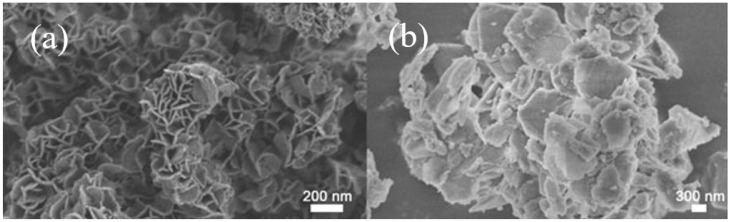
SEM image of (**a**) MgAl LDH and (**b**) CaAl LDH.

Figure 3 presents SEM micrographs of MgAl-LDH and CaAl-LDH. Morphological analysis revealed that MgAl-LDH particles (300–500 nm) exhibit petal-like structures formed by aggregated nanosheets, whereas CaAl-LDH displays well-defined hexagonal platelets (~1 μm diameter; ~20 nm thickness), whose perfect geometric morphology and micron-scale dimensions construct a robust scaffolding framework. The ordered interlayer stacking not only provides ideal interfacial spaces for CaO dispersion but also ensures efficient mass transport due to the nanoscale thickness. Particularly noteworthy is that the excellent geometric regularity of CaAl-LDH crystals effectively inhibits particle sliding during cyclic operations and significantly reduces the agglomeration and sintering tendency of CaO induced by volumetric changes during adsorption–desorption cycles. MgAl-LDH displays a distinctive petal-like nanostructure that exhibits significantly improved three-dimensional spatial distribution relative to CaAl-LDH, indicating strong potential for exceptional structural stability maintenance under operational conditions.

**Figure 4:**
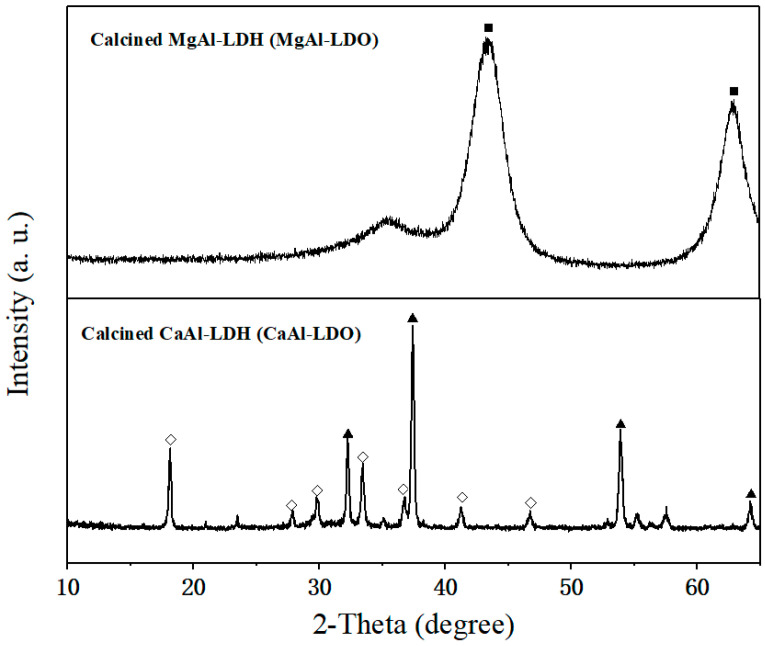
XRD pattern of CaAl-LDH after calcination under 750 °C: (◇) Ca_12_Al_14_O_33_, (▲) CaO, and (■) MgO.

Upon calcination, both CaAl-LDH and MgAl-LDH were transformed into their respective metal oxide forms, as evidenced by the XRD results (Figure 4). CaAl-LDH was converted into a mixture of CaO and calcium aluminate (Ca_12_Al_14_O_33_, JCPDS 09-0413), while MgAl-LDH produced periclase-type MgO with dispersed Al_2_O_3_. As a well-established inert support for CaO-based sorbents, Ca_12_Al_14_O_33_’s efficacy critically depends on its dispersion uniformity within the composite [40]. Conventional methods introduce nanoscale Al_2_O_3_ into CaO matrices to construct support frameworks that inhibit sintering and maintain CO_2_ diffusion channels. Notably, these Al_2_O_3_ additives function directly as physical barriers without forming calcium aluminates (e.g., Ca_9_Al_6_O_18_ or Ca_12_Al_14_O_33_) [41]. In contrast, the atomic-scale homogeneity of Ca/Al in CaAl-LDH precursors enables in situ formation of well-dispersed Ca_12_Al_14_O_33_ phases within the CaO matrix during calcination. This unique structure is expected to significantly enhance both the thermal stability and morphological uniformity of the material.

**Figure 5:**
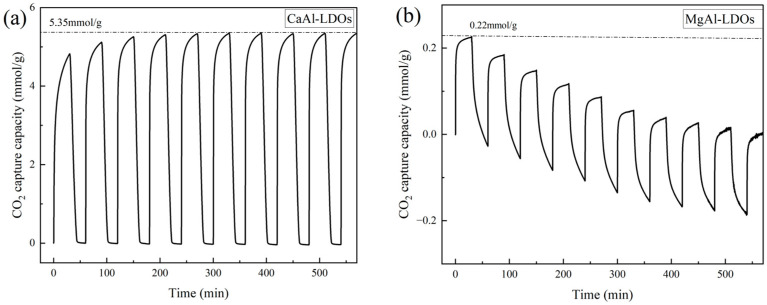
TGA curves of (**a**) CaAl-LDOs and (**b**) MgAl-LDOs.

To evaluate the cyclic stability of derived CaAl-LDOs and MgAl-LDOs, CO_2_ adsorption–desorption cycling tests were performed under 700 °C. The resulting adsorption–desorption profiles are presented in Figure 5. At 700 °C under pressure swing adsorption conditions, MgAl-LDOs exhibit relatively low CO_2_ adsorption capacity; the research conducted by Ke Bian et al. [42] also demonstrated that the CO_2_ adsorption capacity of MgAl-LDOs is not significant. However, as these materials primarily function as supports, their absolute adsorption capacity is not the principal performance metric. Crucially, MgAl-LDOs undergo progressive mass loss during cycling, indicating continuous compositional and structural degradation. This observation demonstrates their poor high-temperature cyclic stability and unsuitability as support materials. In contrast, CaAl-LDOs display higher adsorption capacity than MgAl-LDOs, though their initial capacity (4.82 mmol/g) remains substantially lower than pure CaO. Notably, their capacity increases during subsequent cycles, reaching a maximum of 5.35 mmol/g. Zhihong Xu et al. [43] found that this phenomenon is due to the formation of a “hard skeleton” structure of CaO after calcination. This structural evolution optimizes pore architecture and exposes additional active sites, thereby enhancing CO_2_ adsorption. Despite this activation effect, CaAl-LDOs’ maximum capacity remains inferior to conventional CaO. This limitation stems from the intrinsically low Ca content in CaAl-LDOs, which is constrained by the fixed Ca/Al molar ratio (2:1) inherent to LDH structures. Since the metal ion ratio in LDHs cannot be substantially altered, directly modifying the LDH composition to enhance the CO_2_ adsorption capacity of CaAl-LDOs presents a significant challenge.

### 2.2. The CO_2_ Capture Performance of CaO

During the adsorption–desorption cycling tests, CaAl-LDOs demonstrated exceptional cyclic stability, although their adsorption activity still has room for improvement. Capitalizing on this advantageous characteristic, this study proposes enhancing the Ca/Al ratio by incorporating external CaO sources, thereby constructing a highly dispersed and uniform Ca_12_Al_14_O_33_/CaO composite system through the combination of LDO and CaO. This composite structure not only significantly improves the cyclic stability of the CaO active component but, more importantly, enables precise control over both the adsorption capacity and stability of the composite material.

The CO_2_ adsorption performance of CaO-based materials exhibits pronounced precursor-dependent variations. To identify optimal candidates for advanced study, inorganic (nitrate and carbonate) and organic (acetate) calcium sources were systematically compared. Precursor compounds, such as CaO, Ca(NO_3_)_2_, CaCO_3_, and Ca(CH_3_COO)_2_ (abbreviated as CaAc_2_), were calcined (750 °C, 5 h) to ensure complete decomposition to CaO. CO_2_ adsorption experiments employed reactivation (700 °C, 1 h, N_2_) to eliminate physisorbed species, followed by 20 min thermal equilibration prior to exposure to CO_2_ at 700 °C.

**Figure 6:**
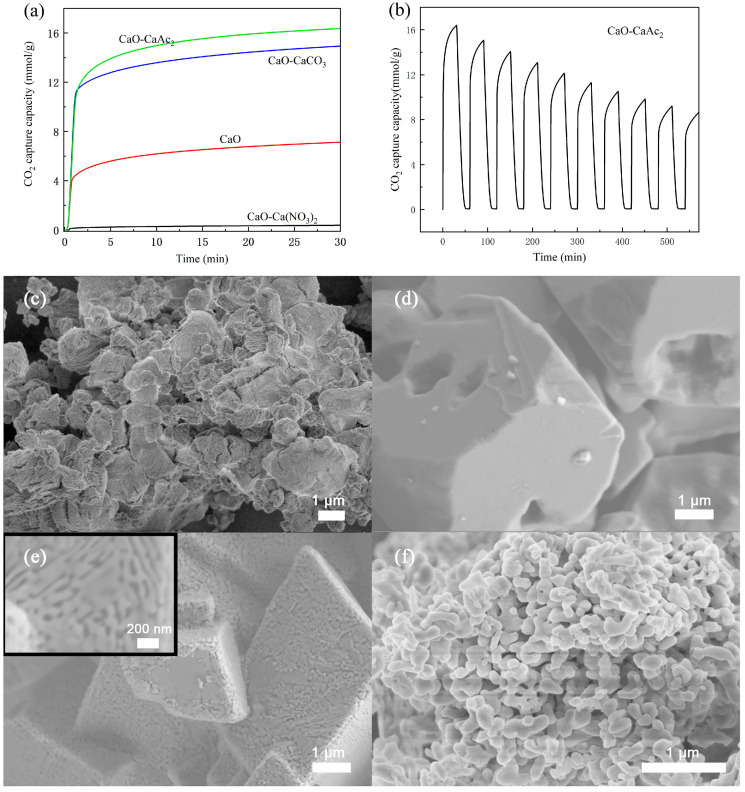
CO_2_ adsorption (**a**) saturation curves of calcium oxide prepared from different precursors, (**b**) cyclic CO_2_ adsorption curve of CaO-CaAc_2_, and SEM images of (**c**) CaO, (**d**) CaO-Ca(NO_3_)_2_, (**e**) CaO-CaCO_3_, and (**f**) CaO-CaAc_2_.

Figure 6a reveals that CaAc_2_-derived CaO demonstrated superior capacity (16.4 mmol/g in 30 min), followed by CaCO_3_-derived CaO (14.9 mmol/g). Pure CaO adsorbed 7.2 mmol/g, while the Ca(NO_3_)_2_-derived material showed negligible uptake (0.4 mmol/g). This performance difference can be attributed to the distinct morphological characteristics observed in Figure 6c–f. Thermal decomposition of organic calcium salts (500–800 °C) produces CaO with enhanced porosity and specific surface area [44], as confirmed by N_2_ physisorption analysis (Table 1). While CaCO_3_-derived CaO showed the highest specific surface area (17.40 m^2^ g^−1^), its large particle size likely hindered adsorption activity. Consequently, calcium acetate was selected as the preferred precursor for subsequent studies. Figure 6b presents the cyclic CO_2_ adsorption–desorption behavior of CaO derived from calcium acetate under controlled conditions. While the adsorbent displayed exceptional initial performance, the absence of structural stabilizers led to progressive sintering-induced degradation, resulting in rapid capacity decay over multiple cycles. The CO_2_ adsorption capacity of CaO derived from CaAc_2_ decreased to 8.2 mmol/g after ten cycles, only half of its initial adsorption performance. The deactivation issue of CaO adsorbents has also been reported elsewhere [45].

### 2.3. Characterization of CaO-CaAl-LDO Composites

**Figure 7:**
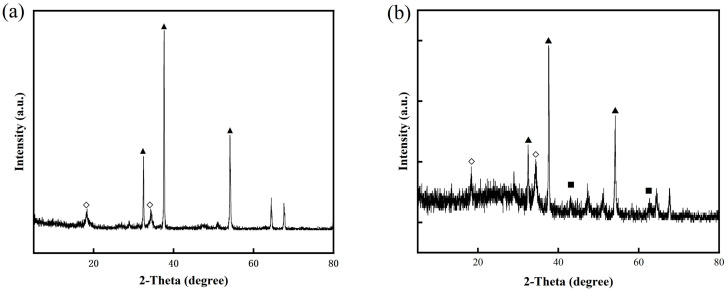
XRD curves of (**a**) CaO-CaAl-LDOs and (**b**) CaO-MgAl-LDOs; (◇) Ca_12_Al_14_O_33_, (▲) CaO, and (■) MgO.

To mitigate CaO sintering, CaAl-LDOs were used as an inert support material. Nanoscale mixing of CaO and CaAl-LDOs was achieved via a solvent/nonsolvent method [46], ensuring uniform dispersion of the CaAl-LDOs within the CaO matrix for effective structural support. Sample characterization was performed to evaluate structural and morphological changes before and after modification. XRD analysis confirmed CaO as the dominant crystalline phase in both CaO-CaAl-LDO and CaO-MgAl-LDO samples, with its sharp diffraction peaks being overwhelmingly predominant, as shown in Figure 7. The Ca_12_Al_14_O_33_ and MgO phases were uniformly dispersed within the CaO matrix as partitioning components, serving as effective anti-sintering barriers to enhance the thermal stability of CaO. Notably, no other calcium aluminate phases (e.g., CaAl_2_O_4_) were detected, confirming the exclusive formation of the desired composite structure.

**Figure 8:**
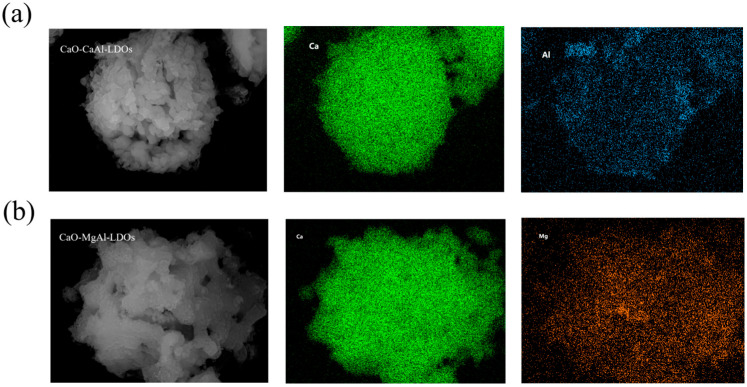
SEM-EDX image of calcined (**a**) CaO-CaAl-LDOs and (**b**) CaO-MgAl-LDOs.

Additionally, scanning electron microscopy–energy dispersive X-ray spectroscopy (SEM-EDX) analysis (Figure 8) quantitatively confirmed the successful synthesis of CaO-dominated composites supported by hydrotalcite-derived scaffolds. In the CaAl-LDH-supported system (Figure 8a), CaO constitutes 93.68 wt% of the material, with residual aluminum (carrier element) reduced to 0.11 wt%, demonstrating near-complete coverage of the hydrotalcite framework by CaO. Similarly, the MgAl-LDH-supported composite (Figure 8b) exhibits 92.04 wt% CaO, while magnesium and aluminum (structural elements of the carrier) persist at trace levels (6.41 wt% and 1.55 wt%, respectively). These results unequivocally establish that both samples achieve >92 wt% CaO loading, a critical threshold for high-capacity CO_2_ capture, while further demonstrating the uniform distribution of hydrotalcite as structural stabilizers, which is essential for mitigating sintering during cyclic carbonation/calcination.

### 2.4. CO_2_ Capture Performance of As-Prepared CaO-CaAl-LDO Composites

**Figure 9:**
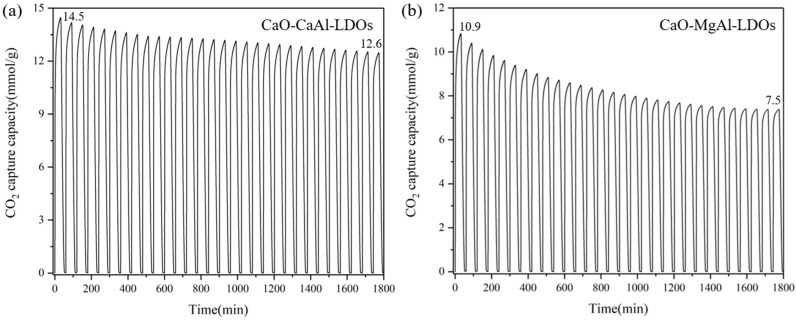
Cyclic CO_2_ adsorption curves of (**a**) CaO-CaAl-LDOs and (**b**) CaO-MgAl-LDOs.

To validate the performance of synthesized CaO-CaAl-LDO composites, CO_2_ adsorption capacity was evaluated by TGA, with the results presented in Figure 9. This enhancement in cyclic stability is principally ascribed to the structural reinforcement imparted by LDH derivatives, which effectively mitigates sintering. Quantitative analysis demonstrates distinct performance differences, where CaO-MgAl-LDOs exhibit progressive capacity deterioration (average of 1.0% decline per cycle relative to initial adsorption), and CaO-CaAl-LDOs maintain superior stability with only 0.4% cyclic attenuation. Notably, both adsorbents achieve substantially enhanced cyclic performance compared to CaO-CaAc_2_, which undergoes severe capacity deterioration (50% loss over 10 cycles). This divergence correlates directly with the inherent thermal stability of LDO supports, as evidenced by CaAl-LDOs’ exceptional high-temperature structural integrity. Consequently, LDH incorporation represents a viable strategy for enhancing CaO’s anti-sintering characteristics, with CaAl-LDH identified as the optimal modifier.

**Figure 10:**
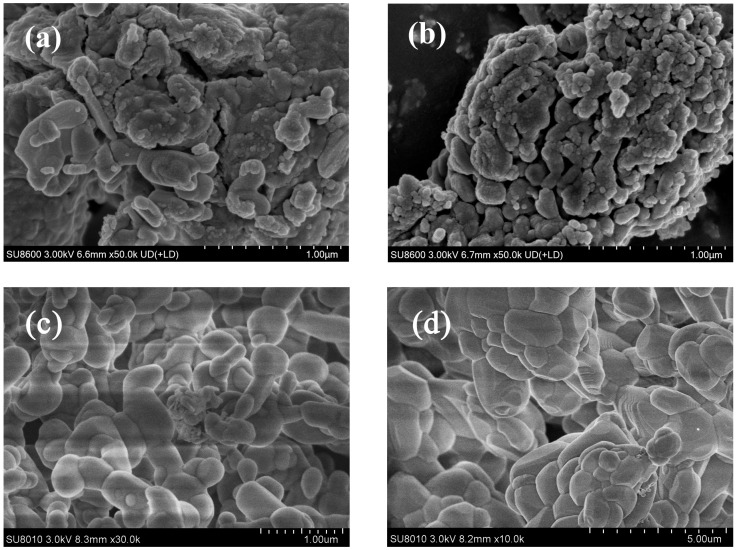
SEM images of (**a**) fresh CaO-CaAl-LDOs and (**b**) fresh CaO-MgAl-LDOs, along with their corresponding morphologies after cyclic CO_2_ adsorption–desorption: (**c**) cycled CaO-CaAl-LDOs and (**d**) cycled CaO-MgAl-LDOs.

Figure 10 demonstrates the critical role of CaAl-LDOs as structural stabilizers through distinct post-cycling morphologies. The solvent/nonsolvent prepared CaO-CaAl-LDO composite (Figure 10c) maintains well-defined hexagonal platelets after 30 cycles, where the formed Ca_12_Al_14_O_33_ phase (XRD in Figure 4) acts as a skeleton that spatially confines CaO nanoparticles within its interlayer domains. This structural confinement effectively anchors CaO particles, mitigating thermal migration and coalescence while preserving mesoporous channels, thereby sustaining efficient CO_2_ diffusion pathways. In contrast, the MgAl-LDO-supported system (Figure 10d) exhibits severe particle sintering and pore collapse due to lattice mismatch and weak CaO–support interactions, resulting in blocked reactive interfaces; this result correlates well with its cyclic stability performance. This result also corresponds to the specific surface area data of the material. After cycling, the specific surface area of the CaO-MgAl-LDOs adsorbent decreased from 13.47 to 9.28 m^2^/g (as shown in Table 2), indicating a certain degree of sintering. The CaAl-LDOs inhibit sintering, which translates to a high retained capacity of 12.6 mmol/g (13% decay from initial CaO). In contrast, the structural degradation of MgAl-LDOs leads to significantly accelerated capacity deterioration, exhibiting a substantially lower capacity retention of 7.5 mmol/g (31.9% decay). Thus, the exceptional stability mechanism of the CaO-CaAl-LDO system originates from the synergistic effects of nanoscale dispersion achieved through the solvent/nonsolvent approach and the thermally stable partitioning framework provided by the CaAl-LDO skeleton. Moreover, since CaO inherently exhibits much higher theoretical adsorption activity than other high-temperature CO_2_ adsorbents, the notable enhancement in cycling stability further enables CaO to maintain higher stable adsorption capacity, making it one of the most competitive high-temperature CO_2_ adsorbents (as shown in Table 3).

## 3. Materials and Methods

### 3.1. Synthesis

Mg_3_Al-CO_3_ LDH was synthesized via co-precipitation. Solution A was prepared by dissolving 0.075 mol of magnesium nitrate (Mg(NO_3_)_2_) and 0.025 mol of aluminum nitrate (Al(NO_3_)_3_) in 50 mL of deionized water. This solution was transferred to a separatory funnel. Solution B was prepared by dissolving 0.05 mol of sodium carbonate (Na_2_CO_3_) in 50 mL of deionized water and placed in a three-neck flask. Under vigorous stirring, Solution A was added dropwise to Solution B. The pH of the reaction mixture was maintained at 10 through the controlled addition of 4 mol/L sodium hydroxide (NaOH) solution. After the complete addition of Solution A, the resulting slurry was stirred overnight at room temperature. The precipitate was then collected by vacuum filtration, thoroughly washed with deionized water until a neutral pH was achieved, and dried at 60 °C for 24 h. The obtained white powder was ground to yield Mg_3_Al-CO_3_-LDH.

Ca_2_Al-NO_3_-LDH was synthesized using a similar co-precipitation method. Solution A contained 0.075 mol of calcium nitrate (Ca(NO_3_)_2_) and 0.025 mol of aluminum nitrate (Al(NO_3_)_3_) dissolved in 50 mL of deionized water, and then it was transferred to a separatory funnel. Solution B was prepared by dissolving 0.05 mol of sodium nitrate (NaNO_3_) in 50 mL of deionized water, and then it was placed in a three-neck flask. Solution A was added dropwise to vigorously stirred Solution B. The pH was maintained at 11.0 during the addition using 4 mol/L NaOH solution. Following complete addition, the mixture was stirred overnight. The product was isolated by vacuum filtration, washed extensively with deionized water to neutrality, and dried at 60 °C for 24 h. The final white powder, obtained after grinding, was identified as Ca_2_Al-NO_3_-LDH.

CaO-CaAl-LDOs and CaO-MgAl-LDOs were synthesized via a solvent/nonsolvent method [46]. An amount of 1.58 g of calcium acetate was dissolved in 10 mL of deionized water until complete dissolution. Subsequently, 20 wt% of Mg_3_Al-CO_3_-LDH or Ca_2_Al-NO_3_-LDH was added to the solution under continuous magnetic stirring to achieve homogeneous dispersion. Upon achieving a clear solution, 10 mL of ethanol was rapidly introduced under vigorous stirring. The resulting gel was transferred to a furnace and calcined at 750 °C.

### 3.2. Characterization of Samples

The materials’ structures were characterized by powder X-ray diffraction (XRD) using Shimadzu XRD-7000 equipment (Shimadzu, Kyoto, Japan) in reflection mode, with Cu Kα radiation and a power of 40 kV × 40 mA (λ = 1.542 Å). Diffraction patterns were recorded from 2θ = 5–80°, with a step size of 0.02°. The materials’ morphology was determined using scanning electron microscopy (SEM, Hitachi SU8600, Tokyo, Japan).

### 3.3. Evaluation of CO_2_ Adsorption Capacity

CO_2_ adsorption capacity was measured using thermogravimetric analysis (TGA55, Waters Technology Co., Ltd., Milford, MA, USA). Layered double hydroxides (LDHs) were pre-calcined (750 °C, 5 h) prior to analysis. Samples were then subjected to pretreatment at 700 °C for 1 h under high-purity N_2_ (40 mL/min) within the TGA. Adsorption was subsequently performed at 700 °C under 100 vol% CO_2_ (1 atm, 40 mL/min) for 30 min, followed by desorption in N_2_ (40 mL/min). Thirty consecutive adsorption–desorption cycles were conducted to simultaneously evaluate adsorption capacity and cyclic stability.

## 4. Conclusions

This study presents a solvent/nonsolvent synthesis strategy to engineer CaO/CaAl-LDO composites, effectively addressing performance degradation caused by sintering in high-temperature CO_2_ capture applications. While calcium acetate-derived CaO exhibits a high initial adsorption capacity (16.40 mmol/g), its irreversible sintering at 700 °C leads to rapid capacity decay. By introducing CaAl-LDO as a nanostructural stabilizer, our method achieves homogeneous dispersion and structural confinement of CaO within the composite, synergistically combining the high adsorption capacity of CaO with the exceptional stability of CaAl-LDO. Remarkably, the composite retains 87% of its capacity after 30 cycles, significantly outperforming both the pure CaO and CaO/MgAl-LDO counterparts. This stability enhancement originates from the formation of a Ca_12_Al_14_O_33_ skeleton during calcination, which suppresses particle agglomeration and preserves hierarchical porosity, thereby ensuring efficient CO_2_ diffusion. The proposed synthesis method is scalable and operationally simple, yielding sorbents that bridge the critical gap between adsorption capacity and cyclic stability.

## Figures and Tables

**Table 1:** BET specific surface area, pore diameter, and pore volume of CaO prepared from different precursors.

Samples	Specific Surface Areas m^2^/g	BJH Pore Diameter nm	BJH Pore Volume cm^3^/g
CaO	7.90	5.16	0.036
CaO-Ca(NO_3_)_2_	3.07	0.55	0.0029
CaO-CaCO_3_	17.40	2.92	0.16
CaO-CaAc_2_	13.50	1.42	0.11

**Table 2:** BET specific surface area, pore diameter, and pore volume of (a) fresh CaO-CaAl-LDOs, (b) fresh CaO-MgAl-LDOs, (c) cycled CaO-CaAl-LDOs, and (d) cycled CaO-MgAl-LDOs.

Samples	Specific Surface Areas m^2^/g	BJH Pore Diameter nm	BJH Pore Volume cm^3^/g
fresh CaO-CaAl-LDOs	13.85	1.24	0.14
fresh CaO-MgAl-LDOs	13.47	1.45	0.09
cycled CaO-CaAl-LDOs	13.82	0.92	0.08
cycled CaO-MgAl-LDOs	9.28	0.85	0.03

**Table 3:** Comparison of CO_2_ capture performance and stability among common high-temperature adsorbents.

**Samples**	**Modification Method**	**Stable Adsorption Capacity**	**Cycling Conditions**	**Reference**
Li_4_SiO_4_	Perlite-derived Li_4_SiO_4_	5.2 mmol/g	100/100% CO_2_	[47]
Li_4_SiO_4_	Fly ash-derived Li_4_SiO_4_	7.7 mmol/g	7/15% CO_2_	[48]
Na_2_ZrO_3_	Wet mixing and heated drying	4.3 mmol/g	8/20% CO_2_	[49]
Na_2_ZrO_3_	ZrSiO_4_-derived Na_2_ZrO_3_	2.5 mmol/g	28/15 CO_2_	[6]
KNaTiO_3_	Rutile sand-derived KNaTiO_3_	3.5 mmol/g	100/100% CO_2_	[50]
CaO	LDO supported	12.6 mmol/g	30/100% CO_2_	This work

## Data Availability

The original contributions presented in this study are included in the article. Further inquiries can be directed to the corresponding authors.
